# Effects of Phototherapy on the Serum Magnesium Level in Neonates with Indirect Hyperbilirubinemia: A Prospective Cohort Study

**DOI:** 10.1155/2022/5439630

**Published:** 2022-03-22

**Authors:** Fatemeh Eghbalian, Somayeh Shabani, Javad Faradmal, Ensiyeh Jenabi

**Affiliations:** ^1^Division of Neonatology, Department of Pediatric, Besat Hospital, Hamadan University of Medical Sciences, Hamadan, Iran; ^2^Department of Pediatrics, School of Medicine, Hamadan University of Medical Sciences, Hamadan, Iran; ^3^Modeling of Noncommunicable Diseases Research Center & Department of Biostatistics and Epidemiology, School of Public Health, Hamadan University of Medical Sciences, Hamadan, Iran; ^4^Autism Spectrum Disorders Research Center, Hamadan University of Medical Sciences, Hamadan, Iran

## Abstract

**Objectives:**

Neonatal jaundice or hyperbilirubinemia is one of the common findings in neonatal medicine. Severe disease can cause neurological damage and even Kernicterus. Magnesium ion is the most important N-methyl-D-aspartate receptor antagonist. The most commonly used treatment for jaundice is phototherapy, but the effect of phototherapy on serum magnesium is less investigated. In this study, we aim to investigate the effects of phototherapy on total serum magnesium levels in icteric neonates.

**Methods:**

This prospective cohort study was carried out on 160 neonates with jaundice referring to the Besat Hospital of Hamadan. Based on the bilirubin level, newborns were divided into three subgroups of mild, moderate, and severe disease which were subjected to single, double, and intensive phototherapy, respectively. Serum bilirubin and magnesium levels were measured before and after phototherapy and compared using parametric tests.

**Results:**

Subjects have a mean intrauterine age of 38.8 weeks and a jaundice onset age of 3.8 days. In all groups, serum magnesium levels were within the normal range before phototherapy. After phototherapy, on the other hand, the most reduction of total serum magnesium was in the double phototherapy group, which was −0.13 ± 0.42 mg/dl (*P* = 0.018). The change in serum magnesium level was not significant in the single phototherapy (−0.02 ± 0.25) and intensive phototherapy (−13.55 ± 2.73) groups (*P* > 0.05).

**Conclusion:**

In the present study, serum magnesium did not increase significantly before the treatment in three groups. After treatment, a significant reduction was seen in the double phototherapy group.

## 1. Introduction

Hyperbilirubinemia is a substantial clinical problem that is the most common cause of newborn hospitalization, especially in Southeast Asia [1, 2]. It refers to an increase in bilirubin levels by >5 mg/dl/day [3]. There are two main types of jaundice in neonates, indirect hyperbilirubinemia (nonconjugated) and direct hyperbilirubinemia (conjunctival). Direct hyperbilirubinemia does not lead to neurotoxicity, while indirect hyperbilirubinemia is toxic and harmful for the brain. When indirect bilirubin reaches a toxic level for neuronal cells, it deposits in the nerve membrane and causes permanent neurological damage to the central nervous system [4–7]. Encephalopathy, Kernicterus, and athetoid cerebral palsy are side effects of the chronic deposition of bilirubin in the neurons of the brain basal ganglia. The high bilirubin in newborns, in contrast to adult jaundice, causes damage to the nervous system, which is related to the lack of complete development of the neonate's blood-brain barrier [4–7]. The most commonly used treatment for hyperbilirubinemia is phototherapy [4–7]. Phototherapy has minor complications including hyperthermia, fever, diarrhea effects on blood cells, cytokines, and vitamins, as well as ocular and dermatological complications [4–7]. Based on Eghbalian and Monsef's studies, phototherapy can lead to hypocalcemia [5] and thrombocytopenia [6] in the newborn. However, phototherapy is generally considered safe [1–4]. The optical energy used in phototherapy reduces the toxicity of bilirubin by converting it to light isomeric or unstable forms of the bilirubin, which have less tendency to fats and can be excreted directly in bile without conjugation [7–9]. However, in cases of severe jaundice that do not respond to phototherapy, transfusion is performed to prevent neurotoxic effects of bilirubin [9, 10]. In the presence of alternative treatment, it can be used in combination with phototherapy to prevent blood transfusion as much as possible. Pharmacotherapy including phenobarbital prescription is another used treatment option, which is not commonly used [9, 10]. In two separate studies, Eghbalian et al. showed that clofibrate could be used as an adjuvant and effective treatment in neonates with indirect hyperbilirubinemia [10, 11].

However, research for other alternative treatments is necessary. Based on studies, bilirubin exerts its toxic effects through the N-methyl-D-aspartate (NMDA) receptor [12, 13]. NMDA is a glutamate receptor and plays a key role in synaptic physiological functions and memory. Bilirubin binds to the neural synapses NMDA and causes overactivation of the receptor, degradation of its ion channel complex in the membrane, and ultimately exertion of its neurotoxic effects [14]. Since magnesium is the main inhibitor of the NMDA receptor in humans, it could play a role in preventing and treatment of hyperbilirubinemia, and even identifying high-risk people and assessing of response to treatment [14]. Magnesium protects the nervous system against hypoxia and neurotoxic effects of bilirubin. It seems to apply these protective effects by blocking the NMDA receptor [14].

Deposition of bilirubin in neurons causes permanent neuronal injury [14]. Bilirubin exhibits an affinity for the phospholipids of plasma membrane-like NMDA receptors. Magnesium is an NMDA antagonist, and it acts against the neurotoxic effects of bilirubin. Several adverse effects have been reported for phototherapy in the treatment of neonatal hyperbilirubinemia but less has been reported regarding the effect of phototherapy on serum magnesium levels [14]. Therefore, this study was carried out to determine the effect of phototherapy on serum magnesium level in term neonates with hyperbilirubinemia.

## 2. Materials and Methods

This prospective cohort study was carried out from March 2017 to September 2018 in the neonatal department of Besat hospital of Hamadan, the capital city of Hamadan province in the west of Iran. The neonatal and NICU department of Besat hospital is a referral center in Iran's western provinces. Healthy term neonates weighing more than 2500 g admitted in the neonatal department due to hyperbilirubinemia with birth age of 2-14 days were included in this study. Before the beginning of the study, written informed consent was obtained from all parents of the children or guardians that were fully informed about the research. Parents of the children or guardians were assured that all their and their child's information would remain confidential in the research group. This project has been approved by the Ethics Committee of Hamadan University of Medical Sciences with the ethics code of IR.UMSHA.REC.1396.123. In the case of abnormality of ABO and Rhesus (Rh), glucose-6-phosphate dehydrogenase (G6PD) deficiency and evidence of any lysis in the tests (hemoglobin reduction, increased retic), history of magnesium sulfate consumption in mother, the use of any medication during admission, and the presence of any signs other than jaundice, the neonates were excluded from the study.

According to a study [15], the reported standard deviation of the magnesium level before phototherapy was 0.36 mg/dl. Considering that the research hypothesis test was able to determine a change of 0.125 mg/dl in the serum level of magnesium after phototherapy with a power of about 80%, therefore, we reached to sample size of 160 newborns. Based on the total serum bilirubin concentration, the newborns were divided into three groups: mild (15-18 mg/dl), moderate (18-20 mg/dl), and severe jaundice (more than 20 mg/dl) [16] with 52, 57, and 51 newborns in each group. They treated with single, double, and intensive phototherapy, respectively. Single phototherapy is phototherapy with a 4-lamp device, double phototherapy has two 3-lamp phototherapy devices on both sides (upper) and at a distance of 15 to 20 cm from the newborn, and intensive phototherapy has eight fluorescent lamps, 4 lamps from the upper part and 4 lamps from the lower part with more than 30 microW/cm^2^/nm.

The basic characteristic and demographic data of the neonates including the age of the jaundice onset (day), intrauterine age (week), weight at admission time (g), delivery type (cesarean section or vaginal delivery), mother's age, total serum bilirubin, and magnesium levels were collected before phototherapy. The method of serum bilirubin and magnesium measuring was photometry and atomic absorption spectrophotometry, respectively. For phototherapy, fluorescence lamps are placed above the head of the neonates with full coverage of the eyes and genitalia. The treatment criterion was the total serum bilirubin levels below 12 mg/dl. Immediately after the completion of the phototherapy treatment period, the total serum bilirubin and magnesium levels of the newborns were measured again.

Before analysis, data were checked and cleaned up and then entered into SPSS version 22. Quantitative variables were described using mean and standard deviation (SD) and qualitative variables using frequency and percentage. To investigate the change in quantitative and qualitative variables, paired-sample *t*-test and McNemar's test were used, respectively. The significance level was considered 0.05 in all cases.

## 3. Results

The subjects were 160 infants with a mean (SD) intrauterine age of 38.76 (0.81) weeks. Ninety-two (57.5%) neonates were born with vaginal delivery. More details were presented in Table 1.

The mean of total serum bilirubin in each of the three groups was presented in Table 2. The amount of total serum bilirubin decreases in all groups.

Table 3 shows that serum total magnesium level in single and double phototherapy decreases after treatment, but this decrease is significant only in the double phototherapy group (*P* = 0.018). In the intensive group, this parameter has slightly increased, which is not statistically significant (*P* = 0.569). In Table 3, the serum total magnesium level and its changes were reported in three groups before and after phototherapy.

The serum magnesium level in newborns before treatment was normal in all three treatment groups (Table 4). The status of each patient showed that 13.2%, 42.1%, and 23.5% of single, double, and intensive phototherapy groups have magnesium content of more than 2.2 mg/dl, respectively.

## 4. Discussion

In this study, total serum magnesium levels in neonates with jaundice were compared before and after phototherapy. For this purpose, 160 neonates with intrauterine age of 38.76 weeks, a mean weight of 3050 g, and a mean jaundice onset age of 3.73 days were evaluated.

In our study, serum magnesium level showed a significant reduction after phototherapy in double phototherapy, but this difference did not show significant changes in both single and intensive phototherapy methods. The reason for insignificant findings in single and intensive phototherapy methods may be a delay in blood sampling due to ethical issues because in our study, no additional blood sampling was performed. Reduced serum magnesium levels after double phototherapy are probably due to increased levels of plasma magnesium in association with hyperbilirubinemia, in which after phototherapy, the magnesium level decreases in association with bilirubin reduction. Since only 1% of the body's magnesium is extracellular, most of these changes are due to the displacement of magnesium between the inside and outside of the cell. Therefore, with increasing bilirubin, plasma levels of magnesium also increase as a result of cellular degradation or as a defense mechanism. In Khosravi et al.'s study, the total serum magnesium levels decreased significantly after phototherapy; it is similar to our results in double phototherapy methods [12].

In a study, Sarici et al.'s reported that in the severe hyperbilirubinemia group, serum ionized magnesium levels were significantly higher in comparison to the moderate hyperbilirubinemia group [13]. But our results revealed that the serum magnesium level was normal in all three groups before the treatment, and there was no increase in serum magnesium level. In Sarici et al.'s study, the increase in magnesium levels in severe hyperbilirubinemia was caused by magnesium leakage from damaged neurons and red blood cells to exert its protective effect on the nervous system [13].

Sapkota et al. reported that phototherapy decreases serum magnesium level as it decreases the serum bilirubin level, and therefore, there is a positive relationship between serum bilirubin and serum magnesium levels, and the rising of magnesium during hyperbilirubinemia may be a physiological compensatory mechanism that counters the toxic effect of bilirubin [14]. In addition, Frargy et al. reported the same relation and recommended oral magnesium supplementation and covering head during phototherapy to prevent reaching light to the pineal gland and so prevention of melatonin decrease which leads to the prevention of hypomagnesemia [17].

In three different studies by Misra et al., Shahriarpanah et al., and Mazary et al., the relationship between hyperbilirubinemia and various minerals was evaluated, total magnesium levels were significantly lower in infants with hyperbilirubinemia compared with the control group, and there was no increase in the serum magnesium level proportional to the increase in serum bilirubin levels [18–20]. These findings were similar to those of our study.

In another study by Karambin et al., most neonates have an intrauterine age of 38 weeks and an average weight of 3221 g, and 90% of infants with jaundice were aged less than five days. They reported similar results in double phototherapy methods, a decrease in serum magnesium levels in response to phototherapy [21]. Our study also had some limitations, including the use of only conventional phototherapy devices, and other devices such as optics phototherapy were not used.

## 5. Conclusion

Severe jaundice can cause neurological damage and even kernicterus due to bilirubin deposition in the membrane of the neurons. Bilirubin exerts its neurotoxicity effect by binding to the NMDA receptor in the neural synapse. Magnesium is one of the most important inhibitors of the NMDA receptor. The body increases the level of extracellular magnesium to reduce the neurotoxicity effects of bilirubin as a defense mechanism. In the present study, the serum magnesium level showed a significant reduction only in the double phototherapy method and remained in the normal range in the other two groups. On the other hand, in all three treatment groups, the level of serum magnesium before the treatment was normal and did not increase significantly.

## Figures and Tables

**Table 1:** Characteristics of neonates for the treatment groups.

Variable	Single		Double		Intensive		*P*
	Mean ± SD	Range	Mean ± SD	Range	Mean ± SD	Range	
Jaundice onset age (day)	3.73 ± 1.82	2-12	3.67 ± 1.56	2-9	3.33 ± 1.47	2-10	0.424^c^
Intrauterine age (week)	38.51 ± 0.62	38-40	38.88 ± 0.93	38-41	38.87 ± 0.81	38-41	0.036^c^
Admission weight (gr)	2981 ± 345	2500-3900	3113 ± 385	2500-4150	3054 ± 363	2500-4000	0.156^c^
Mother's age (year)	28.97 ± 4.77	17-39	27.58 ± 5.32	16-40	29.76 ± 6.24	16-42	0.111^c^
	*N* (%)		*N* (%)		*N* (%)		*P*
Delivery type NVD^a^	25 ± 48.0		34 ± 59.7		33 ± 64.7		0.254^d^
CS^b^	27 ± 52.0		23 ± 40.3		18 ± 35.3		

^a^Normal vaginal delivery. ^b^Cesarean section. ^c^One-way analysis of variance. ^d^Chi-squared test.

**Table 2:** Total serum bilirubin levels before and after single, double, and intensive phototherapy.

Phototherapy types	Serum total bilirubin (mg/dl)		Differences (mg/dl) (Mean ± SD )	*P*
	Before (Mean ± SD )	After (Mean ± SD )		
Single	16.43 ± 0.8	8.60 ± 1.43	−7.83 ± 1.56	<0.001
Double	18.49 ± 0.75	8.82 ± 1.46	−9.67 ± 1.65	<0.001
Intensive	22.80 ± 2.39	9.25 ± 1.25	−13.55 ± 2.73	<0.001

^a^Paired-sample *t*-test.

**Table 3:** Serum magnesium levels before and after single, double, and intensive phototherapy.

Phototherapy types	Serum magnesium (mg/dl)		Differences (mg/dl) (Mean ± SD )	*P*
	Before (Mean ± SD )	After (Mean ± SD )		
Single	2.01 ± 0.28	2.00 ± 0.31	−0.02 ± 0.25	0.547
Double	2.20 ± 0.39	2.06 ± 0.36	−0.13 ± 0.42	0.018
Intensive	2.01 ± 0.31	2.03 ± 0.29	0.02 ± 0.31	0.569

^a^Paired-sample *t*-test.

**Table 4:** Serum magnesium status before and after phototherapy in three treatment groups.

Phototherapy types	Before phototherapy			After phototherapy			*P*
	<1.5	1.5-2.2	<2.2	<1.5	1.5-2.2	<2.2	
	*N* (%)	*N* (%)	*N* (%)	*N* (%)	*N* (%)	*N* (%)	
Single	0 (0.0)	46 (86.8)	7 (13.2)	0 (0.0)	43 (81.1)	10 (18.9)	0.581
Double	1 (1.8)	32 (56.1)	24 (42.1)	3 (5.3)	44 (77.2)	10 (17.5)	0.003
Intensive	1 (2.0)	38 (72.0)	12 (23.5)	1 (2.0)	39 (76.5)	11 (21.6)	0.967

^a^McNemar test.

## Data Availability

The data will be available on request through a data access committee, institutional review board, or the authors themselves.
